# Probiotics and Their Effect on Surgical Wound Healing: A Systematic Review and New Insights into the Role of Nanotechnology

**DOI:** 10.3390/nu13124265

**Published:** 2021-11-26

**Authors:** Alexandra Bekiaridou, Eleni Karlafti, Ilias Marios Oikonomou, Aristidis Ioannidis, Theodossis S. Papavramidis

**Affiliations:** 1 1st Propaedeutic Department of Internal Medicine, Medical School, Aristotle University of Thessaloniki, AHEPA Hospital, 54636 Thessaloniki, Greece; ampekiaridou@gmail.com (A.B.); linakarlafti@hotmail.com (E.K.); i.m.oikonomou@gmail.com (I.M.O.); 2 1st Propaedeutic Surgical Department, University Hospital of Thessaloniki AHEPA, Aristotle University of Thessaloniki (AUTH), 54621 Thessaloniki, Greece; ariioann@yahoo.gr

**Keywords:** surgical wound healing, probiotics, wound dressing, nanotechnology

## Abstract

Skin tissue repair is of fundamental importance for maintaining homeostasis regulation, protection barrier, absorption, and excretion of skin tissue. Wound healing is a complicated process that can be impaired by infections and therefore have a significant economic and social impact. Simultaneously, the overuse of antibiotics has led to antimicrobial resistance and loss of their efficacy. Thus, the need for alternative antimicrobial agents is urgent. The newest approaches on wound dressings employ new therapeutic agents, such as probiotics. Probiotics alone or in tandem with nanotechnology-based techniques exhibit a broad range of benefits on surgical wounds. This systematic review aims to consider current knowledge of probiotic effects on animals and humans regarding surgical wound healing and provide new insights into the role of nanotechnology. The databases included were PubMed (MEDLINE), Scopus, and Cochrane Library (CENTRAL). Studies focused on burns, chronic wounds, and diabetic ulcers were excluded. The promising industry of probiotics demonstrates a significant upsurge as more and more healthy individuals rely their well-being on alternative medicine. Included probiotics illustrated positive results on wound re-epithelization, neovascularization, and wound healing. No adverse effects were noted.

## 1. Introduction

Skin has its own microbiome, which constitutes the skin barrier and is of utmost importance for maintaining human life, providing significant protection from external threats while enhancing homeostasis [1]. After operative procedures, skin continuity is disrupted and follows a complex, protective mechanism of the skin, with the wound healing [1]. Generally, the phases involved in the wound healing process are hemostasis, inflammation, proliferation, and remodelling. A severe wound site infection caused by endogenous flora, including antibiotic-resistant pathogens, can hinder this process. Globally, surgical site infection rates have been reported to range from 2.5% to 41.9% [2]. *Escherichia coli*, *Staphylococci*, and *Enterococci* have been identified as the predominant causes in postoperative wounds [3].

Surgical wound care comes with many challenges, such as patient’s comorbidities and environment, as well as complications related to the standard care protocols. The widespread antimicrobial agents for preoperative prophylactic administration have led to decreased postoperative wound infections. Antibiotic resistance, though, is regarded as the Achille’s heel in surgical wound prognosis. Developing new efficient antibiotics to combat resistant pathogens has been stalled due to economic and regulatory obstacles [4]. Thus, this ongoing need for adequate therapeutic approaches against infections has turned the scientific interest to alternative solutions, such as probiotics, bacteriocins, and nanoparticles [4].

Based on the evidence so far, gut microbiota constitutes an essential factor on the “microbiome–gut–brain axis”, transmitting critical signals to the brain via the vagus nerve. Supplements like probiotics can enhance the microbiome environment via the upregulation of the neuropeptide hormone oxytocin. This hormone alteration has a regulatory role on the hypothalamus and pituitary gland, influencing mammalian homeostasis. The effects on physical and mental health are speculated to be crucial [5].

Probiotics are nonpathogenic microorganisms extracted naturally from various sources like dairy foods [6]. The rationale of their use for medical purposes goes back in time and is based on the hypothesis that oral or topical probiotic administration might replenish the damaged human microbiome. They are promising biomaterials exerting a broad range of positive effects on the human body against pathogens, from gastrointestinal diseases to atopic dermatitis, by stimulating the immune response or directly outcompeting pathogens [7]. Probiotics primarily affect the phase of inflammation, which plays a significant role in wound healing impairment. When applied topically or systematically, recent studies on humans and animals demonstrate a clear-cut benefit on wound healing, affecting the inflammatory response in an oxytocin-mediated fashion. Bacteriocins are bacterial antimicrobial peptides produced by gram-positive and gram-negative bacteria [6]. They are bioactive peptides exerting antimicrobial effects against a broad spectrum of microorganisms. Clinical data must substantiate their safety and efficacy in health conditions. Alternative strategies have been emerging lately, including the integration of nanoparticles and nanotechnology in the development of adequate therapies against wound infections.

In this regard, this review evaluates the effect of probiotics on surgical wounds in human and animal studies. Furthermore, it demonstrates the results of in vitro studies and explores the application of nanotechnology-based wound dressings combined with probiotics.

## 2. Materials and Methods

### 2.1. The Registration and Design

Each step of this systematic review development was based on the PRISMA (Preferred Reporting Items for Systematic Review and Meta-analysis protocols) guidelines (Table S1). Studies that analyzed the association of probiotics and surgical wound healing on animals, humans, and in vitro are included. Two authors searched independently for English literature review. A search for studies published until September 2021 was performed in PubMed (MEDLINE), Scopus, and Cochrane Library (CENTRAL) databases. In case of any disagreement between the examiners, the opinion of the third researcher was taken.

### 2.2. The Assessment of Eligibility and Inclusion

Basic keywords used in search strings were “probiotics”, “bacteriocins”, “wound healing”, “wound dressing”, and “wounds” in both free text and Medical Subject Headings (MeSH) format. Additionally, to define the union between the terminologies, the Boolean operators “AND”, “OR”, and “NOT” were used. The selection criterion was randomized and controlled clinical trials carried out in humans, animals, and in vitro. Articles including burns, diabetic ulcers, and chronic wounds were excluded. Finally, the reference list of the selected studies was manually searched for additional papers not previously identified to enrich the research by retrieving possibly relevant articles. Data were extracted into a table describing the protocol and outcomes of the eligible studies (Table 1 and Table 2). From each selected publication, information about the main author’s name, year, country, population characteristics, intervention, probiotics used, control group, and main results were extracted.

To assess the quality of the 34 studies included in the systematic review, the Jadad scale was used [38]. It consists of five questions related to methodological quality (Table S2). The following criteria are evaluated: whether the trial is randomized and double-blind; whether a description of exclusions and dropouts is detailed; and finally, if the randomization and double-blind method are adequate. A score of 5 points corresponds to the maximum quality level, whereas a score of <3 points is considered to indicate poor quality.

## 3. Results

The literature search identified 34 studies (total sample size, *n* = 133) that investigated probiotic’s effects on human, animals, and in vitro. The basic details of the studies included are presented in Table 1 regarding human and animal studies and Table 2 regarding in vitro studies. Human studies were limited as there were only two [8,9]. Τhere were 23 studies exploring the probiotic effects on animal wounds and nine studies assessing the impact of probiotics on bacteria in vitro. The probiotic tested on humans was Streptococcus thermophilus, and on animals were: *L.* (*Lactobacillus*) *fermentum*, Kefir with *Leuconostoc* spp.; *L. lactis*, *Acetobacter* spp.; *Saccharomyces cerevisae*, *Kluyveromyces marxianus*, *K. lactis*, *L. brevis*, *L. plantarum*, enterocin E3, nisin, clausin, amyloliquecidin, lysostaphin bacteriocins from *L. plantarum* and *L. rhamnosus*, thiopeptide bacteriocin micrococcin P1 (MP1) from *S. hominis* S34-1, bacteriocin BMP32r from *Escherichia coli*, bacteriocin garvicin KS, and *L. chungangensis* CAU 1447. No research has detected possible side effects and the formulations used have also been tested in preliminary studies. The studies included in this systematic review were conducted mainly in small samples of participants, animals, strains, and cultures. The techniques, formulations, and doses of probiotics used were heterogeneous.

A well-treated wound is characterized histologically by a large number of fibroblasts, collagen, and neovascularization. Optimal wound healing involves rapid hemostasis; appropriate infiltration of inflammation factors; mesenchymal cell differentiation, proliferation, and migration to the wound site; neovascularization; prompt granulation; and re-epithelialization [9]. An ideal therapeutic approach could intervene and modulate different phases of wound healing. Probiotics and their effects have been thoroughly studied in vitro and in vivo.

### 3.1. In Vitro Studies

In vitro models mimicking mechanisms of cutaneous wound healing have explored the effectiveness of a probiotic variety, including *Streptococcus thermophilus* extracts, Plantaricin A, and bioengineered nisin.

Firstly, *Streptococcus thermophilus* extracts on human keratinocytes increased hydroceramide and non-hydroceramide levels in keratinocyte cultures, enhancing the lipid barrier [8]. Plantaricin A enhanced the migration of human keratinocyte cells and affected the levels of transforming growth factor-1 (TGF-1), keratinocyte growth factor 7, vascular endothelial growth factor, and interleukin-8 (IL-8) [27,31,39]. A speculated mechanism suggests that the upregulation of IL-8 is relevant to wound healing, as the increase in inflammatory cytokines triggers the wound healing cascade [39]. Another probiotic that demonstrated interesting results was nisin, especially when combined with nanoparticle techniques. Nisin-phosphorylated soybean protein isolate/poly(l-lactic acid)/ZrO_2_ membranes could rapidly decrease *S. aureus* concentrations, as well as a broad spectrum of gram-positive pathogens [18]. The same results were also extracted in trials with bioengineered nisin derivative M17Q. It efficiently inhibited *S. aureus* and *S. epidermidis* biofilms, two ubiquitous pathogens on human skin that constitute a growing threat for surgical wound healing [33]. Tavakolian et al. showed that nisin and lysozyme were predominantly active against gram-positive bacteria but not against gram-negative [35]. Furthermore, Thapa et al., employing a broad combination of peptides that constitute garvicin KS, created a potent combination of garvicin kA and garvicin kB [34]. It emerged as a stable, safe, and efficient antimicrobial agent, but careful testing for a suitable topical formulation applied in human skin is warranted.

Studies conducted on cultures of mouse and human colon cells depicted the effect of probiotics on antiapoptotic mechanisms. They activated antiapoptotic Akt/protein kinase B and inhibited activation of the pro-apoptotic p38/mitogen-activated protein kinase by tumor necrosis factor-α (TNFα), IL-1α, or interferon γ (IFNγ) [37]. Generally, it has been demonstrated that probiotics inhibit apoptosis by affecting cell signaling, enhancing survival.

### 3.2. Animal Studies

#### 3.2.1. Inflammation, Prevention of Infection and Biofilms

The first phase of wound healing includes inflammation, of which the hallmark is the influx of inflammatory cells, including macrophages and polymorphonuclear leukocytes (PMN) [8]. For successful wound healing to be achieved, bacterial counts must be below 10^5^ organisms per gram of tissue and void of any beta-hemolytic Streptococcus bacteria [14]. Accumulating evidence suggests that probiotics interact with the host and/or bacterial cells and inhibit infection-secreting signaling factors [37]. Probiotics act as immunomodulators, and their multiple compounds in various extracts exert pleiotropic effects on wound healing. Wounds treated with Lactobacilli showed a continuous augmentation of PMNs. Lactobacilli attracted neutrophils and macrophages in the wound site by increasing cytokines and chemokines (TNF-α, IFN-γ, IL-4, IL-6, TGF-β, and matrix metalloproteinases) [23,40]. Results were the intensification of the inflammation process and the acceleration of re-epithelialization [24]. Regarding infection prevention, *L. fermentum* and its biosurfactants inhibited *S. aureus* development and the adhesion of bacteria in surgical implant surfaces in vitro [41].

Bacteriocins exhibited multiple ways of action against pathogens. They permeated the cell membrane, destroying cell integrity, while others damaged the bacterial genome irreversibly [31]. Enterocin E3 and bacteriocin from L. plantarum were effective against *S. aureus* biofilms and gram-positive pathogens [10]. Nisin, clausin, amyloliquecidin, and MP1 by *S. hominis* reduced the bacterial loads of *S. aureus*-infected wounds [7,15]. The bacteriocin lysostaphin had lytic effects, decreasing *S. aureus* colonization but not completely eradicating it [19]. Moreover, treatment with recombinant bacteriocin BMP32r led to membrane disruption, intracellular material outflow, and even cell lysis [39].Bacteriocin MP1 and garvicin KS demonstrated broad-spectrum antimicrobial activity against MRSA and many other gram-positive pathogens common in skin infections, including coagulase-negative staphylococci and *E. faecalis* [26]. Interestingly, the combination of rifampicin and MP1 could overcome the isolates resistant to rifampicin and fucidin synergistically [28].

#### 3.2.2. Increase of Re-Epithelization and Re-Vascularization

Probiotics have demonstrated in multiple animal models the ability to improve vascularization and epithelization. Studies focused on the benefits of kefir showed that it increased collagen levels and capillary vessels when wounds were examined microscopically [30]. Wounds treated with kefir had earlier normal tissue reconstruction than the non-kefir group [16]. The increased granulation tissue exhibited abundant neovascularization [11]. In the study of Halper et al.; Lactobacilli injected in wound area promoted the proliferation of newly formed blood vessels accompanied by a few inflammatory cells in the injection site [12]. Additionally, nisin, clausin, and amyloliquecidin led to significant neovascularization and cell migration, aiding in forming a thick epithelial layer [13,15]. *L. brevis* and *L. plantarum* also led to significantly increased fibroblasts and, therefore, collagen production [37].

### 3.3. Human Studies

The effect of probiotics on humans is under-researched in terms of wound healing. To our knowledge, two relevant human studies were conducted by DiMarzio et al. evaluating the stratus corneum sheets in healthy individuals’ forearms and in vitro human keratinocytes [8,38]. The topical application of *Streptococcus thermophilus*-containing cream led to a significant increase of stratum corneum ceramide levels. The results suggested that the cream improved the barrier function and maintained the stratum corneum flexibility. An additional major responsibility of the stratum corneum is the antimicrobial barrier, which serves as a protection against infection by microbial organisms. Ceramide levels of stratum corneum have been associated primarily with skin diseases, and little is known about their effect on wound healing. Nevertheless, research suggests that hydrocolloid dressing containing ceramide-2 promotes regeneration of the epidermal/dermal layers and reduces wound size in the animal in vivo studies [25].

### 3.4. Nanoparticle-Based Techniques in Conjunction with Probiotics

The latest research focuses on nanotechnology combined with probiotics. There are various reasons for this upsurge of interest. Nanoparticle-based technology has improved treatment delivery; nanoparticles ameliorate moisture absorption and retention of substances while having anti-infective properties [42]. Controlled release of entrapped or encapsulated molecules is an essential property of nanofiber wound dressings. This addition can help overcome some limitations of the already-used treatments [17,20].

To our knowledge, the main probiotic used in combination with nanotechnology on wound healing is nisin [18,33]. Nisin has been grafted to a carbohydrate-based nanoparticle, the hydroxypropyl chitosan. Chitosan is characterized by its antimicrobial activity, biodegradability, and biocompatibility [32,43]. It has been utilized to successfully encapsulate nisin with a 95% entrapment efficiency [44]. It has been proven effective against skin infections caused by *S. aureus* and prevented biofilm formation [20,27,36,42]. Furthermore, the conjugation of nisin with silver nanoparticles in nanofibers consisting of poly(ethylene oxide) and poly(d,l-lactide) was also explored. Results were that it inhibited a broad spectrum of gram-positive and negative bacteria over a prolonged period of time [18,27]. The controlled release of probiotics provided by nanoparticles and nanofibers is crucial for maintaining the infection under control [44]. The antibacterial properties of nanoformulated bacteriocins enclosed in nanoliposomes were also optimistic. However, they were not employed in surgical wound infection models. Probiotics were promising synergistic components to the currently used nanoparticles to overcome the emergency of bacterial resistance, but were mainly explored in vitro. Thus, the use for dermatological purposes demands further testing in humans.

## 4. Discussion

Our study addresses the body’s response to the intervention on the “microbiome-gut–brain–axis” by systemic and topical probiotic administration on humans and animals. Furthermore, we explored the effect of the probiotics against pathogens in vitro and their promising role on wound dressings application. This review points to a new potential of probiotic species as integrated therapeutic agents against surgical wound infections.

The development of a wound dressing or probiotic ointment should ensure its efficacy, stability, and safety. Nanotechnology-based techniques, such as encapsulation along with nanoparticles, could facilitate the delivery of treatment. Creating a suitable strategy for probiotic manufacture is an important research project for industrial production, and it must consider the viability and stability of the organisms involved. In the included studies, no adverse effects emerged. Ιn literature, there have been reported bloating, constipation, and thirst when probiotics were administered systematically. Theoretical risks that include systemic infections and excessive immune response have been described without observational data [45,46].

The present research had some limitations. First, there were variations and heterogeneity in the populations included in the different studies, introducing bias. Human studies were only two, while in vitro and in vivo studies employed various techniques and different probiotic doses and types of administration. The strengths of this study are that only randomized clinical trials were included to achieve the highest degree of evidence. Additionally, the review consists of recent studies and provides specific strains, the probiotics used, and the clear-cut outcomes.

## 5. Conclusions

In conclusion, probiotics used in the above studies had various beneficial actions on wounds; they inhibited pathogens, mitigated the risk of infection, and accelerated wound healing. No side effects were observed. Cicatrizing properties of the probiotics themselves and their derived products were not systematically described. This study highlights the potential postoperative benefits of probiotics, demonstrating their multifaceted role in wound healing. Nevertheless, it indicates the urgent need for further research. There is significant heterogeneity of the studies, including the dosage of probiotics administered and the treatment approach. A systematic protocol in human studies, further qualitative research, and testing of potential wound dressings are warranted.

## Figures and Tables

**Table 1:** Detailed summary of human and animal studies included. All the outcomes noted are significantly different from the control groups applied in every study.

Study	Year	Country	Target Area	Treatment	Probiotics Studied	Summary of Key Findings	Animal/ Human Study	Control
DiMarzio [8]	1999	Italy	Forearm skin	Base cream as vehicle containing *S. thermophilus*	*S. thermophilus* extracts	Significantly increased skin stratum corneum ceramide levels	Human	Base cream
DiMarzio [9]	2008	Italy	Forearm skin	Base cream as vehicle containing *S. thermophilus*	*S. thermophilus* extracts	Increased skin ceramides Significantly higher hydration values were found Improved the lipid barrier	Human	Base cream
Gan [10]	2002	Canada	Surgical implants	Solutions with biosurfactant from *Lactobacillus*	*L. fermentum* RC-14	Significantly inhibited *S. aureus* infection Inhibited bacteria adherence to surgical implants	Animal	Negative control group treated with PBS only
Atalan [11]	2003	Turkey	Wounds	Mixture of vaseline and kefir	Kefir	Enhanced wound healing	Animal	Mixture with vaseline
Rodriguez [12]	2005	Brasil	Wounds	Kefir gel	Kefir with *Leuconostoc* spp.; *L. lactis*, *Acetobacter* spp., *Saccharomyces cerevisae*, *Kluyveromyces* *marxianus*, and *K. lactis*	Enhanced wound healing measured by size and histology Improved granulation and neovascularization	Animal	Negative control group treated with 0.9% NaCl Positive control group treated with 5 mg/kg of neomycin–clostebol
Halper [13]	2008	Georgia	Wounds	Subcutaneous injection of lyophilized *Lactobacillus* supernatant	*Lactobacilli*	Stimulated inflammatory stage of tissue repair, TNF-a production, and angiogenesis	Animal	Group treated phosphate- buffered saline in 2% methylcellulose
Zahedi [14]	2011	Iran	Wounds	Ointment with 10^10^–10^11^ CFU/mL bacteria and eucerin	*L. brevis* *L. plantarum*	Significant reduction in inflammation Acceleration of wound healing in wounds treated with *Lactobacilli*	Animal	Untreated negative control group Group treated with eucerin
Zahedi [14]	2011	Iran	Wounds	Ointment with 10^10^–10^11^ CFU/mL bacteria and eucerin	*L. brevis*	Increased number of myofibroblasts Faster decreased inflammation cells Accelerated wound healing	Animal	Untreated negative control group
David [15]	2011	Nigeria	Surgical skin lesion	Gauze soaked in partially purified enterocin E3	Enterocin E3 from *Enterococcus faecalis*	Enterocin E3 was effective against *S. aureus*, *Klebsiella pneumoniae*, *Enterobacter cloaca*, *Listeria* monocytogenes, and *Proteus vulgaris*	Animal	Group treated with distilled water
Nasrabadi [16]	2011	Iran	Full-thickness wound	Mixture of *Lactobacillus* culture with eucerin	*L. plantarum*	Significant reduction in neutrophils, macrophages, and fibroblasts Significant decrease in inflammation Acceleration of re-epithelialization and re-vascularization	Animal	Positive control treated with eucerin Negative control group left untreated
Jones [17]	2012	Canada	Infected wounds	gNO dressings with microbeads containing *L. fermentum* 7230 and sodium nitrite (30 mM)	*L. fermentum*	Increased wound closure Histologically improved healing	Animal	Control patches with glucose (10% *w*/*v*), NaCl (0·85% *w*/*v*) and no sodium nitrite
Heunis [18]	2013	South Africa	Infected wounds	Antimicrobial nanofiber wound dressing	Nisin	Maintained its antistreptococcal activity in vitro for at least 4 days Remained active, even after storage of the formulation at 4 °C for 8 months Significantly reduced the colonization of *S. aureus* in a murine excisional skin infection model Induced an almost complete wound repair	Animal	Nanofiber wound dressings without nisin
Van Staden [19]	2016	South Africa	Infected wounds	Treatment with 12.5 μL (250 μM) of Amyloliquecidin, clausin, or nisin applied directly onto the wound	Nisin from *L. lactis*, Clausin, Amyloliquecidin	Significantly reduced the bioluminescence of *S. aureus* to a level similar to mupirocin treatment Reduced the bacterial load Enhanced wound closure and epithelialization	Animal	Mupirocin-based ointment
Zhu [20]	2017	China	Two strains of bacteria (*S. aureus*, and *Bacillus subtilis*)	Mixture with 50 mL sodium acetate buffer solution, 1.0 g hydroxypropyl chitosan, 1.1 g nisin, 0.25 g of Microbial transglutaminase powder	Nisin	Antibacterial activity against *S. aureus* Antibacterial properties against gram-positive bacteria Improved moisture absorption Promoted cell growth Good antioxidant activity	Animal	Hydroxypropyl chitosan blank control sample
Fu [21]	2017	China	Mandibular Fracture	Injection containing bacteriocin	Bacteriocin isolated from *L. plantarum* ATCC 8014	Bacteriocin could significantly reduce the formation of biofilms and inflammation factor	Animal	Group injected with 1 mL sterile saline solution
Fu [22]	2018	China	Mandibular fracture	Injection containing bacteriocin	Bacteriocin from *L. rhamnosus* L34	Serum levels of TNF-a and CRP were significantly lower than in controls Significantly reduced the formation of biofilms and inflammation of mandible fractures after internal fixation	Animal	Group injected with 1 mL sterile saline solution
Ong [23]	2019	Malaysia	Full thickness wound	A 10% (*v*/*w*) formulated ointment containing 50 μL of the protein-rich fraction from *L. plantarum* USM8613 with 500 mg of soft yellow paraffin	*L. plantarum*	Inhibited *S. aureus* growth Enhanced cytokines and chemokines, wound contraction, keratinocyte migration	Animal	Placebo-treated control group
Xu [24]	2019	China	Infected femoral Fracture with Internal Fixation	Injection with tea polyphenols and bacteriocins	Bacteriocin from *L. plantarum* ST8SH	Effectively controlled *S. aureus* infection	Animal	Negative control treated with saline
Mouritzen [25]	2019	Denmark	Wounds	Mixture of 25 μg/mL Nisin A, 100 ng/mL LPS, or a combination of Nisin A and LPS and incubated at 37 °C, 5% CO_2_	Nisin A from *L. lactis* and lipopolysaccharide	Dampened the effect of lipopolysaccharide and proinflammatory cytokines	Animal	Positive control were cells treated with free amino acids in the same mole-ratio as in Nisin A Negative control left untreated
Liu [7]	2020	China	*S. aureus* infected wound	PEG-PCL-MP1 formula	MP1 from S. hominis S34-1	Reduced *S. aureus* local and systemic infection	Animal	Negative control group MRSA- infected/PEG-PCL
Cheleuitte-Nieves [26]	2020	France	Infected cranial implant margins with MRSA	Liquid lysostaphin (5 mg/mL; total 3 mL/dose) applied topically	Bacteriocin lysostaphin	Decrease MRSA infection short-term, with no resistance discovered	Animal	Systemic administration of antibiotics
Qiao [27]	2020	China	Wounds	Treatment with PBS, 1 × MIC BMP32r (27.6 mg/L) Or 2 × MIC BMP32r (55.2 mg/L)	BMP32r from *E. coli*	Promoted wound healing by killing the multidrug-resistant *S. aureus*	Animal	Negative control group left untreated
Ovchinnikov [28]	2020	Norway	Wounds	Mixture containing 5 mg/mL garvicin KS, 5 mg/mL Penicillin G, and 0.1 mg/mL MP1 in 5% hydroxypropyl cellulose	Bacteriocin garvicin KS and MP1	Efficient in eradicating the MRSA from treated wounds Effective against gram-positive pathogens, such as coagulase-negative staphylococci and *E. faecalis*	Animal	Group treated with Fucidin cream
Nam [29]	2021	Korea	Wounds	100 µL of heat-killed Lc. chungangensis CAU 1447 combined with a eucerin ointment	*L. chungangensis* CAU 1447	Beneficial effects on wound healing	Animal	Negative control group left untreated Positive control group treated with 100 µL PBS)/wound area/day
Ovchinnikov [30]	2021	Norway	Wounds	MP1 (10 µg/mL) in base cream	MP1	Synergistic effects against MRSA Efficiently removed the pathogen from infection sites Prevented its recurrence and resistance development	Animal	Negative control left untreated Positive control treated with fucidin cream

Abbreviations: Enterocin E3, *Enterococcus faecalis* E3; *L. Lactis*, *Lactobacillus Lactis*; *L. Brevis*, *Lactobacillus brevis*; *L. Plantarum*, *Lactobacillus plantarum*; *L. fermentum*, *Lactobacillus fermentum*; *L. Rhamnosus*, *Lactobacillus rhamnosus*; MP1, Τhiopeptide bacteriocin micrococcin P1; PBS, phosphate-buffered saline; BMP32r, Bacteriocin BMP32r; *E. coli*, *Escherichia coli*; MRSA, Persistent methicillin-resistant *S. aureus*; PEG-PCL, glycol and polycaprolactone; *Lc. Chungangensis*, *Lactococcus chungangensis*.

**Table 2:** Detailed summary of in vitro studies included. All the outcomes noted are significantly different from the control groups applied in every study.

Study	Year	Country	Target Area	Treatment	Probiotics Studied	Summary of Key Findings	Control
DiMarzio [8]	1999	Italy	Human keratinocyte cell line	Sonicated bacteria (1.7 g per 5 mL) mixed with 20 mL of a base cream	*S. thermophilus* extracts	Increased ceramide levels	Base cream
Pinto [31]	2011	Italy	Human keratinocyte cells	Co-culture between *L. plantarum* DC400 with *L. sanfranciscensis* DPPMA174 as well as PlnA and hyaluronic acid	Plantaricin A synthesized by *Lactobacillus plantarum*	Promoted wound re-epithelization and neo-vascularization	Basal serum free medium
Jiang [32]	2014	China	Agar culture	Nisin-loaded phosphorylated soybean protein isolate/poly (l-lactic acid)/zirconium dioxide nanofibrous membranes	Nisin	Displayed well-controlled release and better antimicrobial activity against *S. aureus*.	Copper with no nanofibrous membrane
Ahire [33]	2015	South Africa	Soft agar (1 % *w*/*v*) plates seeded with 10^5^ CFU/mL of each bacterial strain (*P. aeruginosa*, *K. pneumoniae*, *S. typhimurium*)	Nanofibers with AgNPs and nisin [silver plus nisin nanofibers (SNF)] Nanofibers containing AgNO_3_ (SF)	Nisin	Inhibited the growth of gram-positive and gram-negative bacteria	Control nanofibers without AgNPs and nisin
Tavakolian [34]	2018	Canada	Bacterial cells	Wound dressings with sterically stabilized nanocrystalline cellulose (SNCC), nisin or lysozyme	Lysozyme, nisin	Effectively inhibited the growth of planktonic *B. subtilis* and *S. aureus* Inhibited the formation of biofilm on microscopy plates Completely killed a 24 h old *S. aureus* biofilm	Unconjugated dressing with lysozyme and nisin SNCC
Mouritzen [25]	2019	Denmark	Human keratinocyte cells Human umbilical vein endothelial cell	25 μg/mL Nisin A	Nisin A	Increased the mobility of skin cells Decreased bacterial growth	Negative control were cells mixed with free amino acids Positive control mixed with epithelial growth factor
Twomey [35]	2020	Twomey	Simulated wound fluid	Agar-based assays with nisin	Nisin A Bioengineered *L. lactis* strains	Significantly reduced the amount of biofilm of *S. epidermidis* formed on all surfaces	Assays without nisin
Peng [36]	2020	China	Erythrocyte solution Murine 3T3 cell cultures	Sodium-type deacylated G–nisin mixture	Nisin bonded with gellan gum (a biocompatible polysaccharide)	The gellan-nisin conjugate kept its antimicrobial properties even with heat alkali treatment at 80 °C or chymotrypsin digestion Showed good biocompatibility Prevented *S. epidermidis* cells from adhering to normal animal cells	Blank control samples without the antibacterial agents Positive control samples with 50 μL of Triton X-100 (1%)
Thapa [37]	2020	Norway	Cultured fibroblast cells	Peptides diluted in solutions	Multi-peptide bacteriocin GarkS from *Lactococcus garvieae* KS1546	Increased overall cell proliferation A combination of two or more antimicrobial agents can have synergistic effects on both non-resistant and resistant bacterial strains	Untreated cells

Abbreviations: *S. aureus*, *Staphylococcus aureus*; *S. epidermidis*, *Staphylococcus epidermidis*; GarKS, Garvicin KS.

## Supplementary Materials

The following are available online at https://www.mdpi.com/article/10.3390/nu13124265/s1, Table S1: PRISMA 2020 statement, Table S2: Assessment of the methodological quality of the included clinical trials, using the Jadad scale.
