# Supplementary material for: Probiotics and Their Effect on Surgical Wound Healing: A Systematic Review and New Insights into the Role of Nanotechnology
Source: Nutrients. 2021 Nov 26;13(12):4265. doi: 10.3390/nu13124265 (PMC8704946; doi:10.3390/nu13124265)
Supplement: Supplementary file 1 [file nutrients-13-04265-s001.zip › nutrients-1472748-supplementary.pdf]

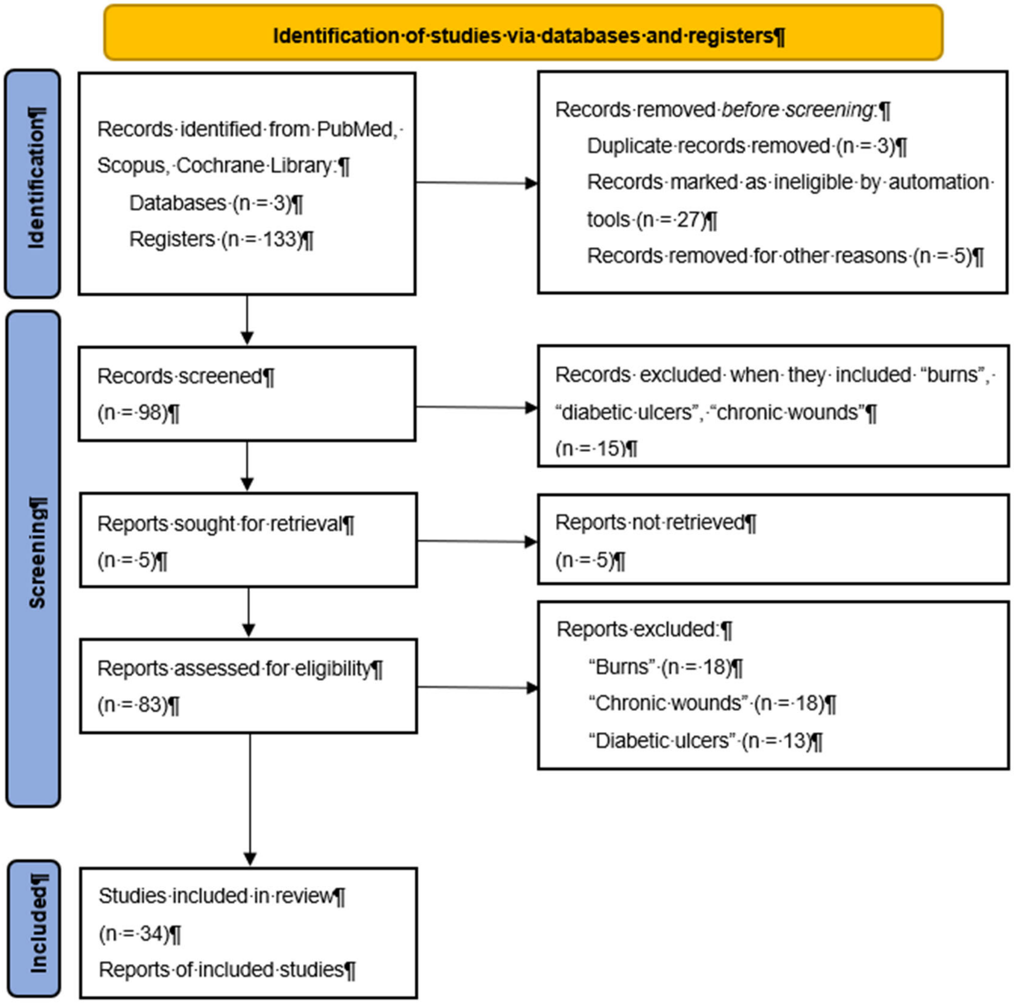

Figure S1. PRISMA 2020 statement.

Table S1. Assessment of the methodological quality of the included clinical trials, using the Jadad scale.

Author	Is the item described randomized?	Is the study described as double blind?	Are exclusions and dropouts of this study described?	Is the method of randomization appropriate?	Is the double-blind method appropriate?	Final score
DiMarzio et al., 1999	1	1	1	1	1	5
DiMarzio et al., 2018	1	0	1	0	0	2
Gan et al., 2002	1	1	1	0	1	4
Atalan et al., 2003	1	1	1	0	1	4
Rodriguez et al., 2005	1	1	1	0	0	3
Halper et al., 2008	1	1	1	0	0	3

Zahedi et al., 2011	1	1	1	0	0	3
Zahedi et al., 2011	1	1	1	0	0	3
David et al., 2011	1	1	1	0	0	3
Nasrabadi et al.	1	1	1	0	0	3
Jones et al.	1	1	1	1	1	5
Heunis et al.	1	0	1	0	0	2
Van Staden et al.	1	0	1	0	0	2
Zhu et al.	1	0	1	0	0	2
Fu et al.	1	0	1	0	0	2
Fu et al.	1	0	1	0	0	2
Ong et al.	1	0	1	0	0	2
Xu et al.	1	0	1	0	0	2
Mouritzen et al.	1	0	1	0	0	2
Liu et al.	1	0	1	0	0	2
Cheleuitte-Nieves et al.	1	0	1	0	0	2
Qiao et al.	1	0	1	0	0	2
Ovchinnikov et al.	1	0	1	0	0	2
Nam et al.	1	0	1	0	0	2
Ovchinnikov et al.	1	0	1	0	0	2
DiMarzio et al.	1	1	1	1	1	5
Pinto et al.	1	0	1	0	0	2
Jiang et al.	1	0	1	0	0	2
Ahire et al.	1	0	1	0	0	2
Tavakolian et al.	1	0	1	0	0	2
Mouritzen et al.	1	0	1	0	0	2
Twomey et al.	1	0	1	0	0	2
Peng et al.	1	0	1	0	0	2
Thapa et al.	1	0	1	0	0	2
